# Editorial: Amino Acids in Plants: Regulation and Functions in Development and Stress Defense

**DOI:** 10.3389/fpls.2021.772810

**Published:** 2021-10-18

**Authors:** Maurizio Trovato, Dietmar Funck, Giuseppe Forlani, Sakiko Okumoto, Rachel Amir

**Affiliations:** ^1^Department of Biology and Biotechnology, Sapienza University of Rome, Rome, Italy; ^2^Laboratory of Plant Physiology and Biochemistry, Department of Biology, University of Konstanz, Konstanz, Germany; ^3^Laboratory of Plant Physiology and Biochemistry, Department of Life Science and Biotechnology, University of Ferrara, Ferrara, Italy; ^4^Department of Plant Pathology, Physiology and Weed Science, Virginia Tech, Blacksburg, VA, United States; ^5^Department of Plant Sciences, MIGAL - Galilee Research Institute, Kiryat Shmona, Israel; ^6^Tel-Hai College, Upper Galilee, Israel

**Keywords:** amino acid metabolism, amino acid transport, stress responses, nitrogen sensing, signaling and assimilation, nitrogen use efficiency, crop enhancement

In the last decades, the importance of amino acids in plant development and stress defense has become increasingly evident, attracting growing interest in basic and applied plant science. Here we present novel findings on amino acid research and propose a picture, as up-to-date as possible, of the current knowledge on this fascinating aspect of plant physiology. Besides being building blocks for protein synthesis, many amino acids, including some not involved in protein synthesis, turned out to have active roles in plant development and participate in the plant's response to environmental stresses. In addition, amino acids serve as precursors for many primary and secondary metabolites and have pivotal roles in human nutrition, either as a source of nutraceutical compounds or as essential dietary components. Indeed, nine out of twenty-one proteinogenic amino acids cannot be synthesized in animals, including human beings, and three or more others are not synthesized in sufficient quantities to satisfy the metabolic needs (Figure 1). These nutritionally essential amino acids must be taken up from the diet and by far the greatest share is derived from plants (Galili et al., 2016, Hou and Wu, 2018). In contrast to humans and animals, plants synthesize all twenty-one proteinogenic amino acids themselves (Figure 1).

**Figure 1:**
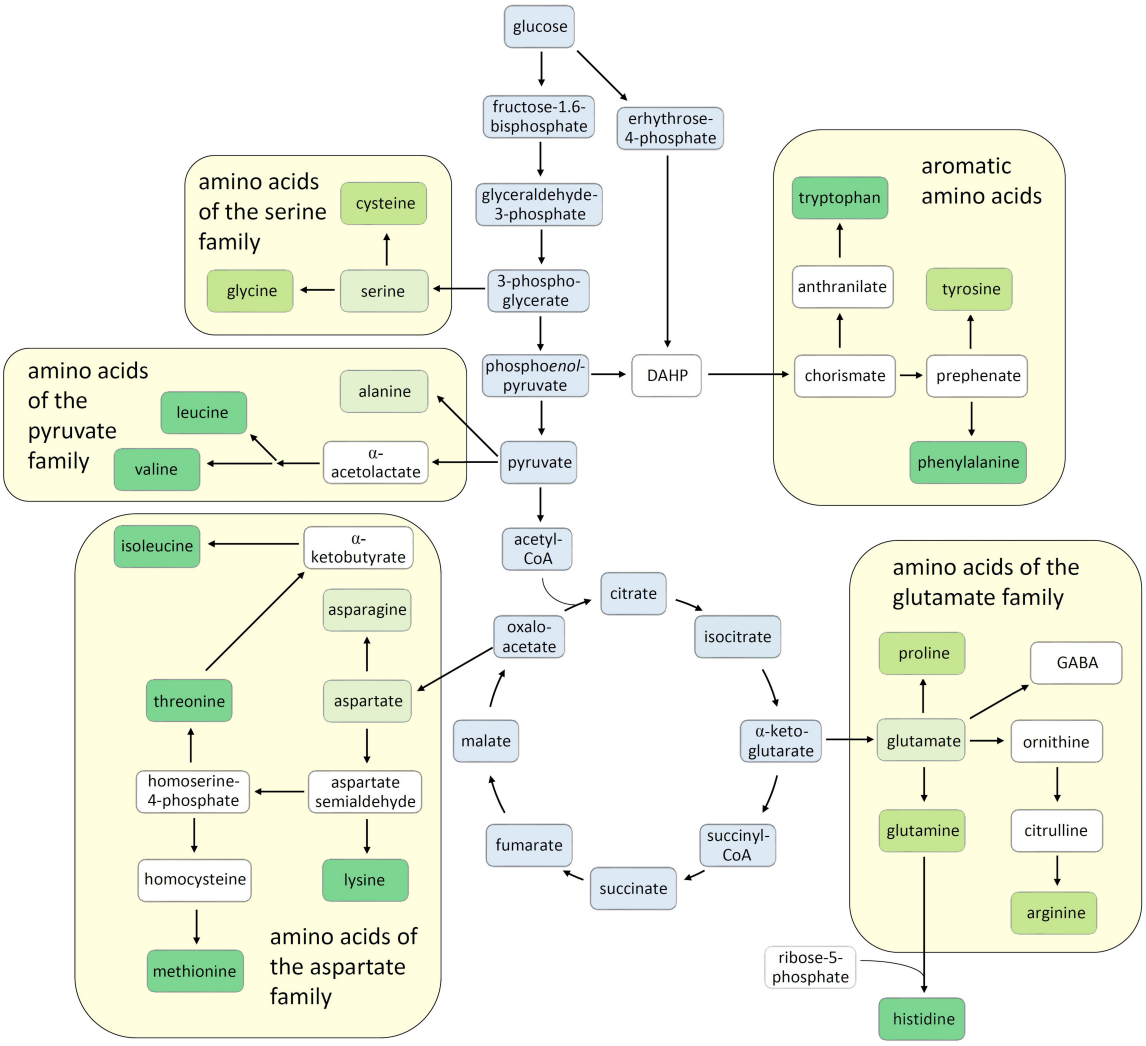
Amino acid biosynthesis in plants. The carbon skeletons of amino acids are derived from different intermediates of the central carbon metabolism (boxed in blue). According to their respective precursors, the amino acids are grouped into five families derived from glutamate, serine, pyruvate, aspartate, or chorismate. The nine amino acids that cannot be synthesized in animals are shown in dark-green boxes, while those that can be synthesized but additionally need to be taken up with the diet are in brighter boxes. Proteinogenic amino acids that can be sufficiently synthesized in animals are in pale green boxes and non-proteinogenic amino acids and other important intermediates are boxed in white. DAHP, 3-deoxy-D-*arabino*heptulosonate-7-phosphate.

Lysine, as one of those essential amino acids is often present at low levels in plants and thus limits their nutritional value. The review of Yang et al. focuses on lysine catabolism and describes the connections between the degradation intermediates of this amino acid and other metabolic pathways, such as tryptophan metabolism, tricarboxylic acid cycle, abiotic and biotic stress responses, starch metabolism, and the unfolded protein response. A closer look at the relationships between lysine and serine is provided by Kavi Kishor et al., who summarize the current knowledge on the biosynthetic pathways, regulatory mechanisms, and biological effects of both amino acids, focusing on the complexity of their interactions. The authors report the complex mechanisms of transcriptional and post-transcriptional regulation and highlight the importance of proteins rich in lysine and serine for plant development and stress tolerance. Furthermore, the role of non-coding RNAs in the regulation of lysine- and serine-rich proteins and the possibility of using genome-wide strategies to identify novel interactions are discussed. While the interest in lysine biosynthesis largely aims at improving the nutritional value of crops, the research on the catabolism of this amino acid focuses more on tolerance to biotic and abiotic stresses. Arruda and Barreto surveyed the known pathways of lysine degradation, with special attention to the saccharopine pathway. As described in the review, the effects of lysine catabolism on stress tolerance are probably due to the production of proline and pipecolate from glutamate and α-aminoadipate-δ-semialdehyde, respectively, mediated by the saccharopine pathway.

Methionine is another essential amino acid intensively studied to increase its content, which is usually very low in crop plants and vegetables. Cysteine can be synthesized from methionine but is still considered nutritionally essential due to the low methionine content in many plant tissues. Whitcomb et al. used a metabolic engineering approach to increase the content of both methionine and cysteine in rice seeds by generating a pair of transgenic lines. Each transgenic line contained a methionine-rich seed storage protein and an exogenous enzyme for either methionine or cysteine biosynthesis. This strategy successfully increased methionine content in seeds by approximately 50% but revealed an unexpected sulfur deficiency-like molecular phenotype and an alteration in seed protein profile, possibly caused by the accumulation of unfolded proteins in the endoplasmic reticulum. A similar approach was used by Girija et al. to investigate whether the overall capacity to synthesize methionine or the density of methionine residues in seed proteins is the limiting factor for methionine content in seeds. The authors' conclusions suggest that the abundance of methionine residues in storage proteins is likely the main factor limiting methionine accumulation in Arabidopsis seeds. Moreover, they confirmed the association between the increase in methionine content and the accumulation of stress-related metabolites in seeds, although the reasons for this correlation remain unknown. The complexity and importance of sulfur-containing amino acids are also tackled by Watanabe et al. who reviewed the metabolism and regulatory functions of O-acetylserine, S-adenosylmethionine, homocysteine, and serine, as essential precursors of cysteine and methionine synthesis.

Besides being essential components of the animal and human diet, amino acids or their derivatives can provide a rich source of nutraceutical metabolites. An example of a health-promoting functional compound is represented by γ-aminobutyric acid (GABA), a non-proteinogenic amino acid whose remarkable properties have been reviewed by Gramazio et al. Additionally, this review summarizes the newest breeding strategies to increase GABA content in crops focusing on new CRISPR/Cas9-based approaches recently used to successfully improve GABA concentration in ripe tomato fruits with no adverse side effects.

Methionine, as well as other amino acids, are also used for the synthesis of glucosinolates, a large class of sulfur-containing metabolites with recognized antioxidant and anticancer properties. Lächler et al. investigated the function of isopropylmalate isomerase, an enzyme essential for leucine synthesis and possibly involved in methionine chain elongation. The active enzyme is a heterodimer composed of a large subunit and one among three possible small subunits. In Arabidopsis, the large protein is encoded by a single gene, while three different genes encode the small subunit. By studying the substrate specificity and the expression patterns of the subunits, the authors found that the large subunit is involved in both leucine and glucosinolate metabolism, and the small subunits appear specific for each pathway. In particular, the small subunit 1 is involved in leucine biosynthesis and the small subunits 2 and 3 function in methionine-derived glucosinolate synthesis.

Besides their role as nutraceutical molecules, many non-proteinogenic amino acids are involved in the plant responses to environmental stresses, as confirmed by Song et al. for citrulline, an intermediate in the synthesis of arginine from ornithine. By transcriptomic and metabolomic analysis, the authors demonstrated that the rapid accumulation of citrulline and related metabolites in watermelon subjected to water stress is mediated by the synchronized activation of biosynthesis and suppression of catabolism. Additionally, they found that the nitrogen status of the plant regulates citrulline synthesis.

Polyamines in plants are generated either from arginine or ornithine. In the former route, arginine is decarboxylated to agmatine by arginine decarboxylase and then converted into putrescine by agmatine iminohydrolase and N-carbamoylputrescine amidohydrolase. In the latter route, arginine is hydrolyzed to ornithine by arginase and then decarboxylated by ornithine decarboxylase to putrescine. It was recently shown, however, that arginases from Arabidopsis and soybean can act also as agmatinases, providing a third route for putrescine synthesis (Patel et al., 2017). To shed light on the mechanism of this reaction, Sekula analyzed, by X-ray and small-angle X-ray scattering, the crystal structures of two arginases from *Arabidopsis thaliana* and *Medicago truncatula* and proposed a model to explain the dual binding properties of plant arginases.

Among the amino acids involved in stress defense, proline is especially important because it accumulates in most plant species in response to different stresses and is believed to contribute to stress tolerance. Proline accumulation largely depends on the transcriptional activation of δ^1^-pyrroline-5-carboxylate synthetase (P5CS), the enzyme catalyzing the rate-limiting step of proline biosynthesis, which in most plants species is encoded by two paralogous genes. As reported by Sabbioni et al., the activity of P5CS2 in rice is additionally regulated by post-translational mechanisms to regulate proline synthesis in accordance with the redox and nitrogen status of the plant cell. In Arabidopsis, different expression patterns of the two P5CS isoforms indicate functions of P5CS1 in stress-induced proline accumulation and stress tolerance and of P5CS2 in proline synthesis for growth and development. Additionally, variable localization of the two isoforms in both the cytosol and plastids was reported (Székely et al., 2008). Funck et al., however, partly challenged these notions, finding that both P5CS1- and P5CS2-GFP fusion proteins were only present in the cytosol and that the contribution of both isoforms to stress tolerance was very low. Surprisingly, these authors found that p5cs2 mutants were more salt-tolerant than either p5cs1 mutants or wildtypes, despite a lower proline content. These results suggest a new function for P5CS2 in salt tolerance and reinforce the hypothesis that proline metabolism rather than proline itself is responsible for stress tolerance. A novel assay for the quantitation of L-proline was reported by Forlani and Funck. This assay is more specific than the widely used ninhydrin method (Bates et al., 1973). According to the authors, ninhydrin-based methods erroneously detect related molecules, such as ornithine, hydroxyproline, and D-proline, and lose linearity in the presence of high amino acid concentrations, resulting in overestimations of proline content. The method proposed by Forlani and Funck, based on the reverse reaction of P5C reductase (P5CR) at unphysiological pH of 10.5, can overcome the specificity limits of the conventional colorimetric methods while maintaining similar sensitivity.

Regardless of whether proline metabolism or proline accumulation confers stress tolerance, circumstantial evidence points to the importance of this amino acid in the reproductive stage and suggests that its accumulation may maintain productivity under stress conditions, as reported in Arabidopsis by Mattioli et al. and in barley by Frimpong et al. Based on previous work (Mattioli et al., 2018), Mattioli et al. confirmed the importance of proline accumulation in pollen grains to maintain seed production under salt stress, although the possibility to further improve grain yield by forcing proline synthesis in pollen grains remains unproven. With a completely different approach, Frimpong et al. analyzed five spring barley genotypes with contrasting responses to drought, including two lines harboring a P5CS1 allele introgressed from a wild barley accession. They found a correlation between proline accumulation and water stress tolerance, particularly in spikes. The lines bearing the wild P5CS1 allele turned out to be the more drought-tolerant at the reproductive stage leading to improved grain yield under water stress. Intriguingly, the beneficial effects of proline under stress may also occur when proline is provided from the outside, as reviewed by El Moukhtari et al., who updated the current knowledge on this topic and proposed possible mechanisms of action. Although we still do not know how exogenous proline can improve salt stress tolerance in crops, this procedure is recognized as an effective method of improving stress tolerance in crops and regarded as of utmost biotechnological interest. A similar approach is reported by Alfosea-Simón et al., who investigated the use of external formulations made up of or enriched in different amino acids to improve plants' resilience to climate changes. By morphological, physiological, and metabolomic analyses, the authors studied the effects of exogenous applications of glutamate, aspartate, and alanine on tomato growth, and found a synergistic and positive effect of aspartic and glutamic acid and a negative effect of alanine.

Proline accumulation during stress relies on both stimulation of proline synthesis and inhibition of proline degradation. The former process is catalyzed in the cytosol by the sequential action of P5CS and P5CR, while the latter is catalyzed in mitochondria by the sequential action of proline dehydrogenase (ProDH) and pyrroline-5-carboxylate dehydrogenase (P5CDH). Because P5CS and ProDH catalyze the rate-limiting steps of proline synthesis and oxidation from and to glutamate, respectively, a careful determination of their activity levels is often used as a marker of proline accumulation. A common mistake in ProDH activity determination was disclosed by Lebreton et al., who showed that, at pH 10, ProDH does not catalyze the proline-dependent reduction of NAD^+^. On the contrary, this activity was attributed to P5CR, which at high, non-physiological pH, is also able to work in the reverse direction.

In addition to proline, various other amino acids have been involved in stress tolerance, among which the branched-chain amino acids (BCAAs) have been recently proposed. Buffagni et al. investigated the role of BCAAs in two durum wheat cultivars with contrasting sensitivity to drought, performing a comparative bioinformatic and expression analysis of the genes coding for BCAA transferases (BCAAT), and investigating, through NMR analysis, the metabolic profile of the BCAAs. Overall, they showed that BCAAT genes are induced transcriptionally in early phases of the stress response, and the accumulation of BCAAs reflects the cultivars' drought tolerance, supporting the involvement of BCAAs in the drought defense response.

In plants, the aromatic amino acids (AAAs) are synthesized from chorismate, the final product of the shikimate pathway, and are precursors of a wide range of secondary metabolites. To investigate a possible role of AAAs in the resistance to biotic and abiotic stress, Oliva et al. generated transgenic tobacco plants overexpressing a feedback-insensitive version of AroG, a 3-deoxy-D-*arabino*-heptulosonate 7-phosphate synthase gene, encoding the first enzyme of the shikimate pathway. A metabolomic analysis confirmed that the leaves of the transgenic plants contained higher levels of phenylalanine, tyrosine, and tryptophan, as well as related metabolites compared to control plants. The transgenic plants gained some resistance to salt stress but not to oxidative or drought stress and strong resistance to infections with the plant parasite *Phelipanche aegyptica*, suggesting that increasing AAA levels in plants can be an effective strategy to combat plant parasites. The shikimate pathway, and thus the synthesis of AAAs, is the target of glyphosate, a herbicide used world-wide. In particular, glyphosate is a competitive inhibitor of the enzyme 5-*enol* pyruvyl-shikimate-3-phosphate synthase (EPSPS). Zulet-González et al. analyzed the fast-growing weed *Amaranthus palmeri*, some populations of which are glyphosate-tolerant because they overexpress EPSPS, to investigate the role of AAAs in the regulation of the shikimate pathway and glyphosate resistance. They found a complex interaction of glyphosate and AAAs in feedback-regulation of the shikimate pathway, which was altered by EPSPS overexpression. The mechanisms underlying this effect, however, remain unknown.

Regardless of the multiple functions of amino acids in plant development and stress defense, amino acids need nitrogen for their biosynthesis, and understanding how nitrogen is taken up, stored, and transported in plants is of utmost interest in amino acid biology. O'Neill and Lee describe a method to determine both the abundance and localization of free amino acids in plant tissues, which can be of great help to address these topics. The authors successfully used matrix-assisted laser desorption ionization (MALDI)- mass spectrometry imaging (MSI), coupled with coniferyl aldehyde derivatization, to study the uptake and distribution of amino acids in the maize root, proposing the use of MALDI-MSI as a valid method to study nitrogen assimilation, storage, and transportation in plants.

Because arginine has a high nitrogen-to-carbon ratio, plants tend to store nitrogen as arginine when nitrogen is abundant. Arginine accumulation is achieved by relieving feedback inhibition of the arginine biosynthesis gene N-acetylglutamate kinase (NAGK). This mechanism depends on the regulatory protein PII, which is able to sense the nitrogen and carbon status of the cell to optimize arginine biosynthetic activity. In a study from Llebrés et al. the structural and functional characteristics of the PII protein from maritime pine are presented, adding new information on the mechanisms of arginine metabolism regulation.

Ammonium is the primary source of inorganic nitrogen used for amino acid synthesis. Ammonium assimilation and recycling require the concerted activity of glutamine synthetase (GS), glutamate synthase (GOGAT), and glutamate dehydrogenase (GDH). While GS and GOGAT are the most important enzymes for the assimilation of organic molecules in plants, GDH participates in glutamate homeostasis and provides the TCA cycle with 2-oxoglutarate (2OG) when carbon availability is limiting. A structural study on GDH1 from *Arabidopsis thaliana*, is presented by Grzechowiak et al. who analyzed the crystal structure of AtGDH1, both in its apo form and when bound to its cofactor NAD^+^ and the reaction product 2OG. The research revealed that the enzyme undergoes an open/closed conformational change, which needs the binding of both NAD^+^ and 2OG for its full activation. Most of the soil ammonium is taken up by the plant members of the high-affinity ammonium transporter (AMT) family, especially AMT1;1 and AMT1;2. Recent evidence (Xuan et al., 2016) showed that treatment with brassinosteroids (BRs) enhances the expression of AMT1;1 and AMT1;2 in rice with a mechanism yet to be understood. To investigate the relationships between AMTs and BRs, Yang et al. studied the levels of AMT expression and the AMT-dependent efficiency of ammonium uptake in rice lines with altered expression of BES1 and BZR1, two master regulators of BR signaling in plants. The authors' conclusions suggest that BR regulation of NH4^+^ uptake in rice involves transcriptional regulation of ammonium transporters and that BR-dependent ammonium uptake is partially controlled by BZR1.

A class of transporters, namely USUALLY MULTIPLE ACIDS MOVE IN AND OUT TRANSPORTERS (UMAMITs), were recently identified as amino acid transporters (Ladwig et al., 2012). A specific function of amino acids to sense the nitrogen availability of the cell and share this information among different pathways to trigger appropriate metabolic responses was addressed by Besnard et al. They studied the overexpression of some UMAMIT genes in Arabidopsis to investigate possible links between amino acid transport and stress responses and found strong evidence that amino acid export activity is positively correlated with stress phenotypes and pathogen resistance, most likely due to the establishment of a constitutive salicylic acid-mediated stress response. The relationships between amino acids and hormonal pathways found by Besnard et al. and Yang et al. and the connections with key metabolic pathways reported by different authors of this collection, highlight the central role of amino acids in stress and development and pave the way to new and exciting discoveries in basic and applied plant science.

## Author Contributions

All authors listed have made a substantial, direct and intellectual contribution to the work, and approved it for publication.

## Funding

MT acknowledges support from Sapienza University (Progetti Ateneo 2020), GF acknowledges support from the University of Ferrara (FAR 2021), SO acknowledges support from National Science Foundation (MCB 1052048, IOS 135336), RA acknowledges support from Tel Hai College and from Israel Science Foundation (1857/20).

## Conflict of Interest

The authors declare that the research was conducted in the absence of any commercial or financial relationships that could be construed as a potential conflict of interest.

## Publisher's Note

All claims expressed in this article are solely those of the authors and do not necessarily represent those of their affiliated organizations, or those of the publisher, the editors and the reviewers. Any product that may be evaluated in this article, or claim that may be made by its manufacturer, is not guaranteed or endorsed by the publisher.
